# Pazopanib radio-sensitization of human sarcoma tumors

**DOI:** 10.18632/oncotarget.24281

**Published:** 2018-01-20

**Authors:** Feng Wang, Hongyan Li, Ela Markovsky, Ryan Glass, Elisa de Stanchina, Simon N. Powell, Gary K. Schwartz, Adriana Haimovitz-Friedman

**Affiliations:** ^1^ Department of Radiation Oncology, Memorial Sloan Kettering Cancer Center, New York, NY, USA; ^2^ Anti-Tumor Assessment Core Facility, Memorial Sloan Kettering Cancer Center, New York, NY, USA; ^3^ Department of Medicine, Division of Hematology/Oncology, Columbia University Medical Center, New York, NY, USA; ^4^ Current address: Albert Einstein Cancer Center, Albert Einstein College of Medicine, Bronx, NY USA

**Keywords:** single high dose radiation therapy (SDRT), pazopanib, tumor angiogenesis, ceramide, apoptosis

## Abstract

Recent data in our laboratory indicate that engagement of host-derived microenvironmental elements impact tumor response to single high dose radiation therapy (SDRT). In these studies we showed that microvascular endothelial damage plays a critical role in tumor response as regulator of direct lethal damage of SDRT. Using a genetic model of Acid Sphingomyelinase (ASMase)-deficient mice we showed that activation of this enzyme by SDRT-induced damage in the endothelium is mandatory for tumor cure. ASMase activation triggers ceramide-mediated apoptosis, and therein microvascular dysfunction, which increased the vulnerability of tumor cells to lethal damage by radiation. Angiogenic factors repressed this activity while a monoclonal antibody targeting VEGF, de-repressed ASMase activity and radiosensitized tumor endothelium when delivered immediately prior to SDRT. In this study, we tested the effect of SDRT in combination with the short-acting anti-angiogenic agent, Pazopanib (anti-VEGFR-1/2/3, PDGF-α/β and c-kit), in two xenograft models of human sarcoma. Pre-treatment with a single dose of Pazopanib increased SDRT-induced ASMase activity and endothelial dysfunction *in vitro* and *in vivo*, enhancing SDRT tumor cure, and exhibiting critical dependence on timing relative to SDRT exposure, suggesting a mechanism of action identical to that demonstrated for anti-VEGF/VEGFR2 antibodies. These results demonstrate the ability of Pazopanib to shift the response towards tumor cure and could therefore have a significant impact on clinical trial development in combination with SDRT for sarcoma cancer patients.

## INTRODUCTION

As one of the potent and efficient cancer therapies, radiation is widely used to treat a variety of cancer types. Ionizing radiation (IR) works by damaging the DNA of normal and cancerous tissue leading to cellular death [1]. The goal of radiation therapy (RT) is to maximize damage to cancer cells while keeping collateral damage to a minimum [1, 2]. Studies in this field revealed that IR-induced DNA damage repair is more efficient in normal cells than in tumor cells, which display dysregulated repair [2]. Standard RT protocols for cancer patients use a low dose (1.8–2.0 Gy) exposure repeated daily until the maximal tolerable normal tissue doses are reached. Given that the total dose delivered to tumors is generally determined by normal tissue toxicity rather than by the curable dose required for tumor eradication, the overall local cure using fractionated approach is ~65% of all tumors, and curability depends on tumor type and size. Following the development of intensity modulation RT (IMRT) and image guidance RT (IGRT), which can improve precision in tumor targeting and reduce normal tissue exposure, SDRT is gradually becoming an alternative therapeutic strategy. SDRT has shown clinical benefit with local control rates at over 90% in a variety of cancer types, including tumors considered resistant to conventional fractionated RT [3, 4]. Although SDRT presents a practical alternative approach for fractionated radiotherapy, its biological mechanism remains elusive. We recently reported that SDRT induces a rapid wave of endothelial cell apoptosis via ceramide generation in both normal gastrointestinal tract and tumors [5–7]. In this regard, SDRT launches a rapid translocation of acid sphingomyelinase (ASMase) to the endothelial plasma membrane, where ASMase converts sphingomyelin to the second messenger ceramide and the latter mediates apoptotic signaling [8, 9]. The 20-fold enrichment of a non-lysosomal secretory form of ASMase results in endothelial cells being particularly vulnerable to radiation-induced ASMase-mediated generation of the pro-apoptotic second messenger ceramide [10–12]. Importantly, our previous studies showed that the angiogenic factors bFGF or VEGF restrain radiation-induced ASMase activation, ceramide generation, and consequent endothelial apoptosis [12, 13]. Conversely, anti-angiogenic agents, such as anti-VEGFR2 antibody, antagonize these effects, synergistically increasing radiation-induced ceramide elevation and enhancing apoptosis [14]. Furthermore, *in vivo* these agents cause a synergistic increase in radiation-induced tumor endothelial apoptosis and enhanced tumor response to SDRT [14]. These studies therefore define that an ASMase/ceramide pathway-dependent endothelial response plays a crucial role in tumor cure by SDRT and is modulated by angiogenic factors.

Tumor angiogenesis, the recruitment of new blood vessels, is essential for tumor growth and metastasis, and is driven by a balance between anti-angiogenic and pro-angiogenic factors [15]. Anti-angiogenic therapy is emerging as an effective treatment for various tumor types through direct targeting of VEGF (such as the antibody bevacizumab) or the inhibition of VEGFRs by multi-target tyrosine kinase inhibitors (TKIs) [16–18]. These anti-angiogenesis strategies interfere with either the development or functionality of the tumor-associated vasculature, and thereafter lead to suppression of oxygen and nutrition supply to cancer cells [17]. Recently, two different concepts have proposed that anti-angiogenic tumor therapy may either ‘‘normalize’’ dysfunctional tumor vasculature, which therefore facilitates drug delivery, or prevent recruitment of circulating endothelial precursors into the tumor [18, 19]. Although the outcomes of some clinical studies support either of these hypotheses, to date anti-angiogenesis therapy has yielded only modest therapeutic gains. The accurate mechanisms remain to a large extent unknown and the lack of an optimized mode of application limits the utility of this approach.

Pazopanib, (GW786034B, 5-[[4-[(2,3-dimethyl-2H-indazol-6-yl)methylamino]-2-pyrimidinyl]amino]-2-methyl-benzenesulfonamide), a novel and potent vascular endothelial growth factor receptor inhibitor [20], is a small-molecule inhibitor shown to target both tumor and endothelial cells in multiple myeloma [21]. Pazopanib targets the TKRs including VEGFR-1/2/3, PDGFRα/β, and c-KIT [22]. Pre-clinical studies have shown that Pazopanib can inhibit tumor angiogenesis and the growth of several human tumor xenografts (multiple myeloma, colon, melanoma, prostate, kidney) in mice [22]. In addition, in 2009 Pazopanib was approved by the US FDA for the treatment of patients with advanced renal cell carcinoma (RCC). Additionally, several recent phase II and III studies have shown a significant clinical benefit of Pazopanib in a variety of malignancies, including soft tissue sarcoma, thyroid cancer, and ovarian cancer [21–23]. In the current study we tested the curative potential of a combination of SDRT with Pazopanib on xenografts of human sarcoma tumors, a chondrosarcoma (JJ012) and a neurofibrosarcoma (MPNST3). Our results revealed that a single dose of Pazopanib mimics the anti-VEGF/VEGFR impact on tumors subsequently exposed to SDRT, increasing ASMase activity in the serum and tumor endothelial dysfunction, enhancing tumor response, and exhibiting critical dependence on timing relative to SDRT exposure. These results suggest that Pazopanib has a similar mechanism of action to the one we previously demonstrated for anti-VEGF/VEGFR2 antibodies. As a short-acting anti-angiogenic, Pazopanib might be optimal for endothelial-mediated radiosensitization, and in combination with SDRT it might allow dose de-escalation, thus significantly expanding the range of clinical indications for SDRT.

## RESULTS

### Pre-treatment of Pazopanib radiosensitized JJ012 and MPNST3 sarcomas

**Figure 1:**
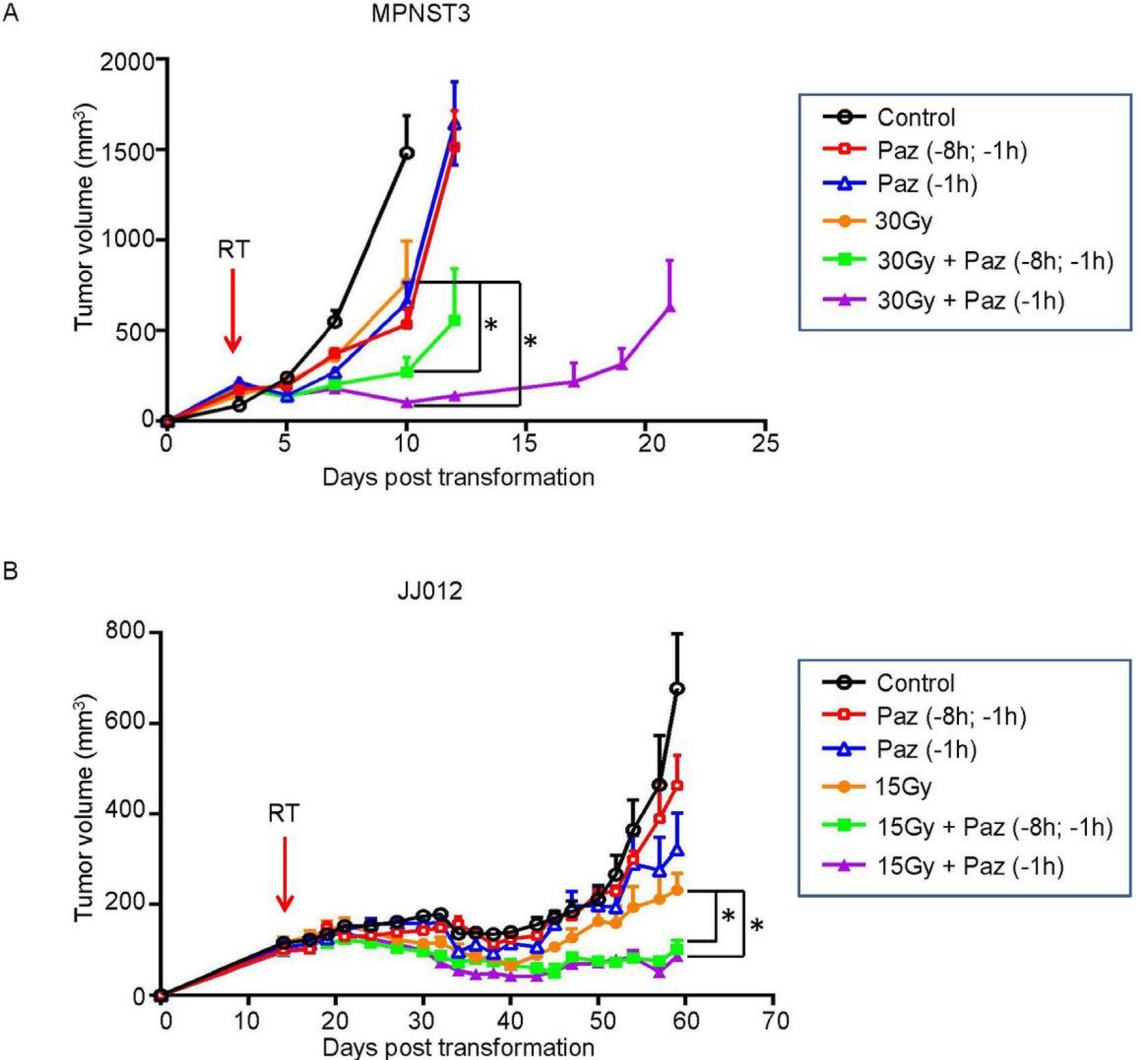
Pazopanib pre-treatment significantly increased tumor response to SDRT in JJ012 and MPNST3 sarcoma tumor models MPNST3 tumor tissue was transplanted subcutaneously into the right flank of ICR-SCID mice (**A**) and JJ012 cells were implanted subcutaneously into the right flank of athymic mice (15 × 10^6^cells/mouse) (**B**). When the tumors reached the volume of ~150 mm^3^, the mice were administered a single p.o. dose of Pazopanib (100 mg/kg) at 1 h or two p.o. doses at 8 h and 1 h prior to 30 Gy or 15 Gy SDRT. The tumor volumes were measured for the indicated days. Volumes are mean ± SEM. Arrows indicate the day of SDRT. (^*^p < 0.05; 30 Gy vs 30 Gy + Paz and 15 Gy vs15 Gy + Paz).

Our previous studies have shown that angiogenic factors protect endothelial cells from radiation-induced apoptotic death, and anti-angiogenics antagonized this effect and increased tumor response [14, 23]. Here we tested the effect of radiation therapy in combination with Pazopanib, a VEGFR inhibitor and a short-acting anti-angiogenic agent, on two mouse models of human sarcoma. Athymic or ICR/SCID mice were transplanted with JJ012 or MPNST3 sarcoma tumors respectively. When tumor volume reached 150 mm^3^ the tumors were treated with IR and/or Pazopanib, and their volumes were measured. As shown in Figure 1A and 1B, Pazopanib alone (single-dose or two-doses) administration resulted in a slight tumor growth delay relative to non-treated control mice in both sarcomas, whereas no significant difference between a single dose (−1 h) or two-doses (−8 h and −1 h) pre-administration cohorts was observed. SDRT (a single dose of 30 Gy) yielded a significant tumor response (*p* < 0.05 vs control) in MPNST3 tumors. Pre-treatment with single-dose or two-doses of Pazopanib prior to SDRT, radiosensitized MPNST3 response and led to enhanced tumor growth delay as compared to SDRT alone (Figure 1A). Notably, single-dose Pazopanib administration resulted in a greater tumor growth delay than in the two-doses Panzopanib administration cohort. A similar result was obtained in JJ012 tumors, SDRT alone (15 Gy) robustly reduced tumor growth, while pre-treatment with Pazopanib (single- or two-dose/s) significantly increased the radiation effect on tumor growth inhibition (Figure 1B, *p* < 0.05 vs SDRT alone). Although sarcoma tumors are modestly radio-responsive, pre-treatment with Pazopanib radiosensitized both JJ012 and MNPST3 sarcoma tumors and improved their response significantly.

### Combination of SDRT and Pazopanib increased tumor endothelial cells (TEC) apoptosis and decreased median vascular density (MVD)

**Figure 2:**
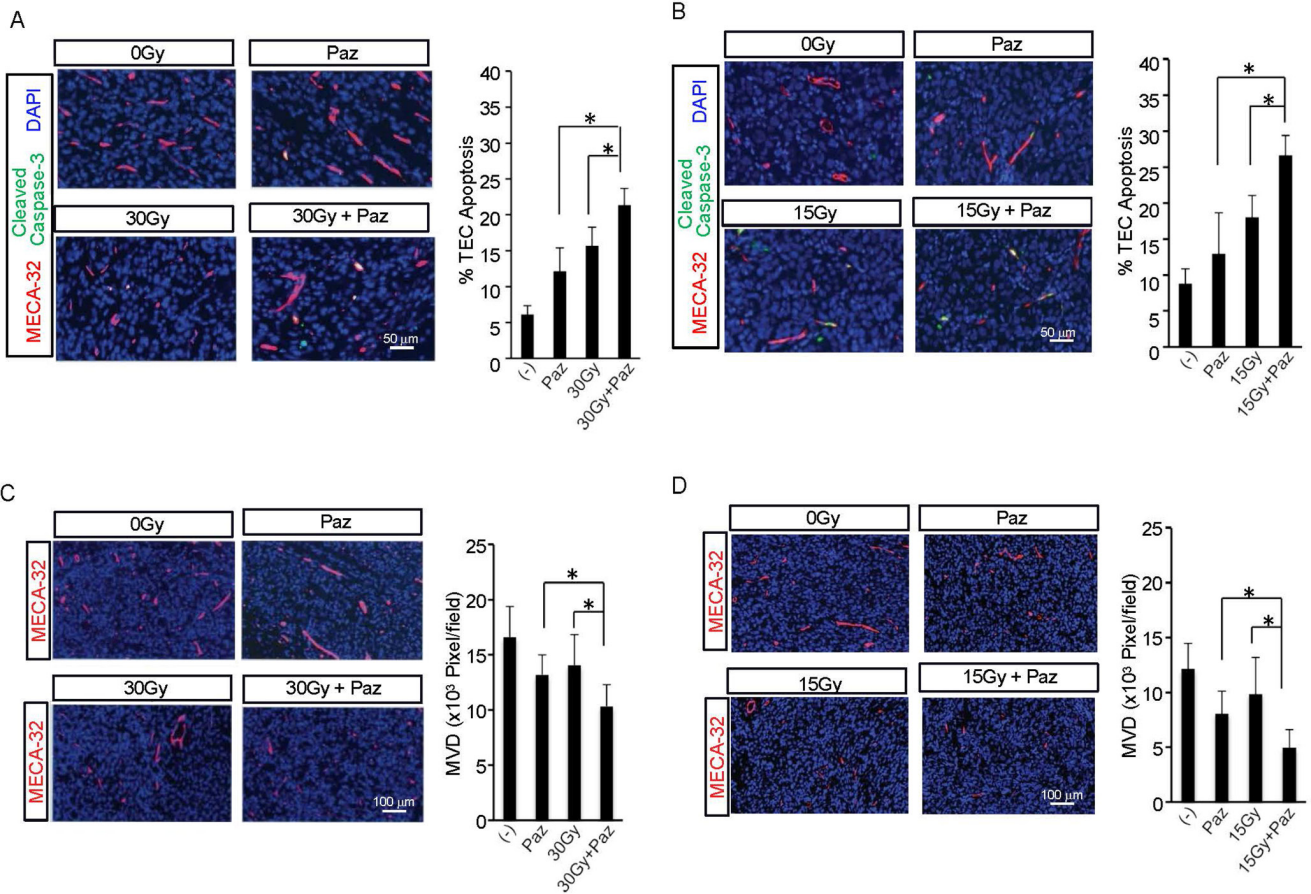
Effect of Pazopanib pre-treatment on SDRT-induced TEC apoptosis and MVD Pazopanib (100 mg/kg p.o.) was administered to MPNST3 bearing ICR-SCID mice (**A, C**) and JJ012 sarcoma bearing SCID mice (**B**, **D**) and after 1 h tumors were irradiated with 30 Gy and 15 Gy, respectively. Tumors were removed 6 h after irradiation, fixed in 4% paraformaldehyde, and embedded in paraffin. 5-mm sections of tumor specimens were double stained with MECA-32 (red), to detect TEC, and cleaved caspase-3 [50], to identify apoptotic cells. Data (mean ± SD) are collected from 5 mice per group with 1000 endothelial cells evaluated. Scale bar, 50 μm. (^*^p < 0.05).

Recent studies from our group and others demonstrated that the *in vivo* apoptotic damage delivered by SDRT to endothelial compartment is critical for SDRT-induced tumor cure, whereas angiogenesis factors (VEGF, FGF etc.) partially reversed this effect [6, 13, 14]. We showed above that Pazopanib enhanced SDRT effect in two human sarcoma tumor models. Next, we determined the effect of the combined therapies on the vasculature. First, we evaluated TEC apoptosis at 6 h post-radiation. JJ012 and MNPST3 tumors were pre-treated with Pazopanib (−1 h, 100 mg/kg p.o.) and irradiated with 15 Gy or 30 Gy accordingly. As shown in Figure 2A and 2B, compared to a baseline TEC apoptosis in non-treated control JJ012 tumors (8.8 ± 2.1%) and MNPST3 tumors (6.1 ± 1.2%), Pazopanib alone generated a modest increase of TEC apoptosis in JJ012 (13 ± 5.6%) and in MPNST3 (12.2 ± 3.2%). While SDRT alone triggered a significant increase in TEC apoptosis (18 ± 3% in JJ012 tumors and 15.7 ± 2.6% in MPNST3 tumors), in the combination treatment with Pazopanib and SDRT there was a further increase in the TEC apoptosis (26.7 ± 2.7% in JJ012 tumors and 21.3 ± 2.3% in MPNST3 tumors), which was significantly higher than each treatment alone. These observations indicated that the combination of Pazopanib and SDRT significantly increased TEC apoptosis in human sarcoma animal models when Pazopanib is administered 1 h before radiation.

Tumor angiogenesis is a critical factor for tumor growth, and MVD is an important parameter for assessing angiogenesis in tumors (14, 15). Subsequently, we quantified the effect of combination of SDRT and Pazopanib on tumor MVD. Figure 2C and 2D show that Pazopanib alone reduced MVD to 56.2% ± 14.5% (vs control) in JJ012 tumors and to 79.5% ± 10.8% (vs control) in MPNST3 tumors. Whereas SDRT alone caused a decrease in MVD in JJ012 to 68.8 ± 23.3% (vs control) and to 84.6 ± 16.8% (vs control) in MPNST3, the combination of SDRT and Pazopanib showed an enhanced decrease in MVD in JJ012 (34 ± 11.8%, vs control) and MPNST3 (62.3 ± 11.8%, vs control). These results indicate that Pazopanib enhances SDRT effect on tumor growth delay by significantly increasing SDRT-induced TEC apoptosis and reducing angiogenesis in these tumors.

### SDRT in combination with Pazopanib decreased tumor perfusion

**Figure 3:**
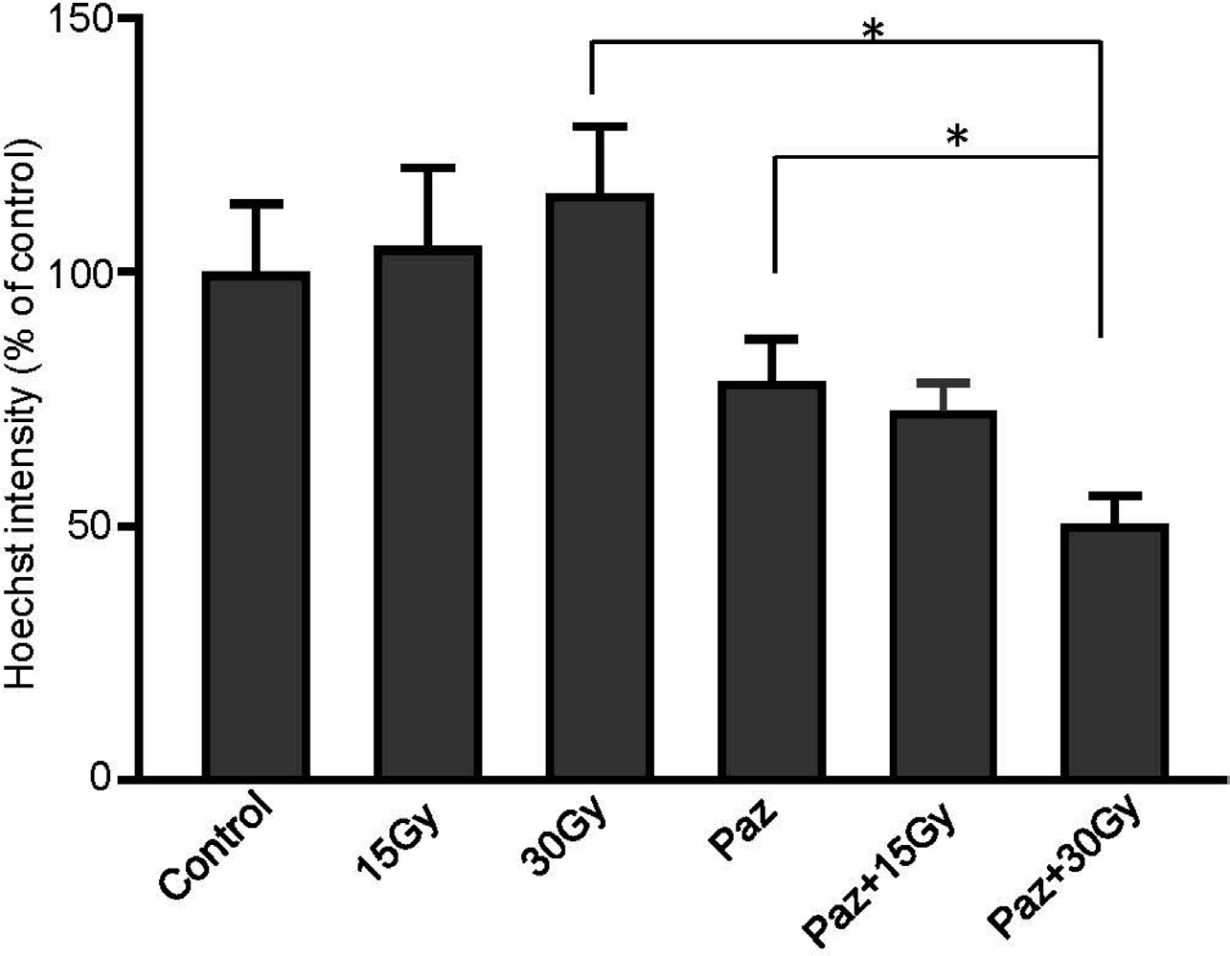
Combination of SDRT with Pazopanib reduced perfusion in MNPST3 tumors Perfusion was measured by injection of Hoechst 33342 fluorescent dye. Irradiation with 30 Gy combined with Pazopanib pre-treatment (100 mg/kg p.o.) resulted in 50% decrease in perfusion in the tumor 30 minutes post RT, compared to the untreated tumors (^*^p < 0.01).

Subsequently, irradiation with 30 Gy combined with Pazopanib pretreatment of MPNST3 tumors resulted in 50% decrease in perfusion in the tumors 30 minutes post SDRT, compared to untreated tumors (Figure 3, *p* < 0.01). Treatment with 15 Gy or 30 Gy with Pazopanib and Pazopanib alone caused a 30% decrease. Radiation alone did not cause a decrease in perfusion in this tumor model. These results indicate that the improved tumor growth delay in response to combination of Pazopanib and SDRT treatment are due to enhanced microvascular dysfunction generated by this combination treatment in these sarcoma tumor models. Similar results were obtained in pre-clinical mouse tumor models using SDRT in which the perfusion reduction was accompanied by secretion of ASMase into the systemic circulation within the first hour of radiation (Campagne and Fuks submitted publication).

### SDRT in combination with Pazopanib increased ASMase activity *in vivo*

**Figure 4:**
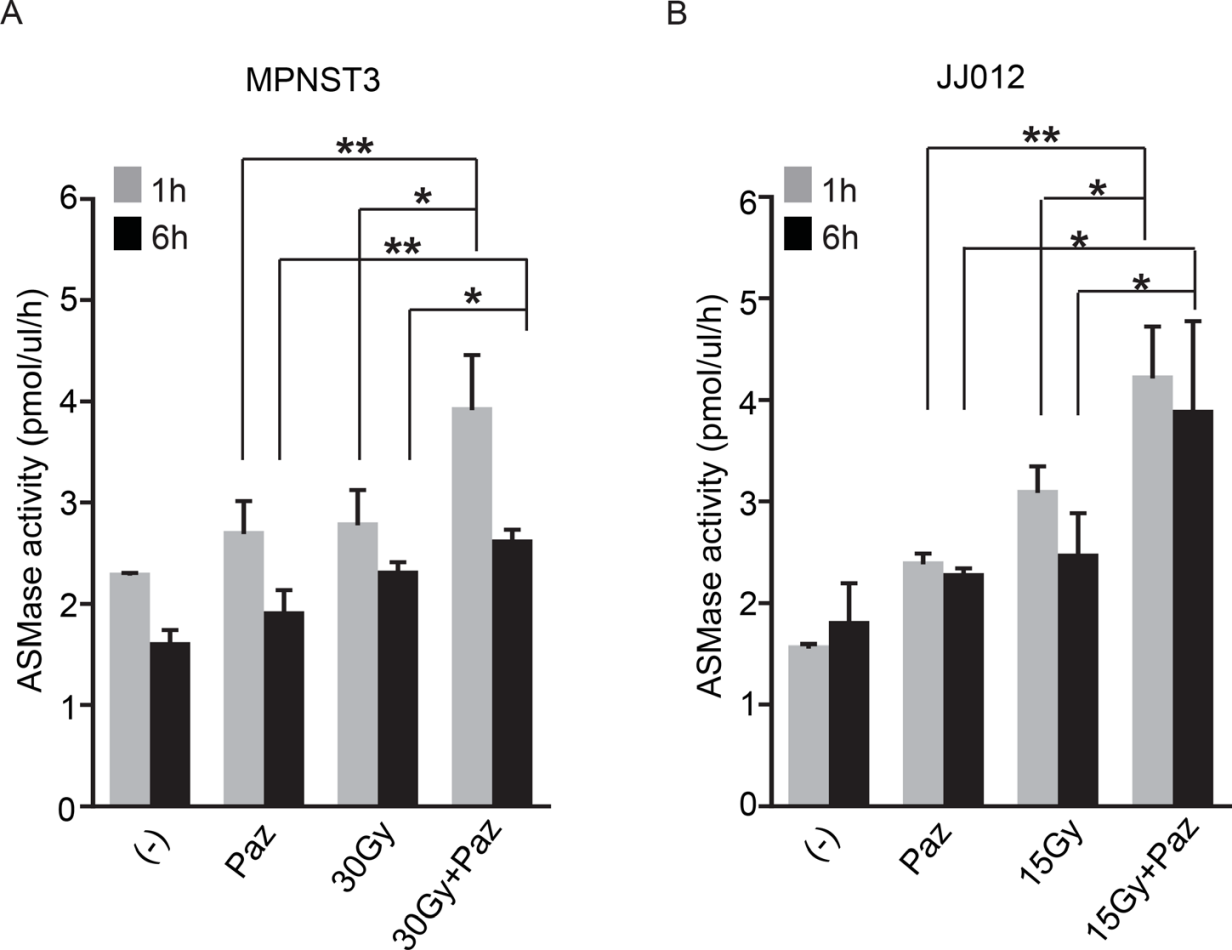
Combination of SDRT with Pazopanib increased ASMase activity MPSNT3 (**A**) and JJ012 (**B**) tumors were irradiated with 30 Gy or 15 Gy after 1 h pre-treatment with Pazopanib (100 mg/kg p.o.). Mouse serum was collected at 1 h and 6 h after radiation and ASMase activity was measured by quantifying conversion of (^14^C)sphingomyelin to the product (^14^C)phosphocholine. Data is mean ± SD. ^*^p < 0.05 and ^**^p < 0.01.

Accumulated evidence establishes that SDRT-induced TEC apoptosis depends on ASMase/Ceramide-mediated signaling *in vivo* [6, 14, 23]. SDRT induces rapid translocation of ASMase to endothelial plasma membrane where it generates ceramide and initiates the apoptotic signal. Next, we evaluated whether the effects of combination of Pazopanib and SDRT on the TEC *in vivo* are mediated via the ASMase activity. JJ012 and MPNST3 bearing mice were treated as described above, and mouse serum was collected at 1 h and 6 h after SDRT. Both Pazopanib treatment and SDRT increased serum ASMase activity at 1 h and 6 h post radiation in both human sarcoma models (Figure 4A and 4B) as compared to baseline control cohorts. Combination of Pazopanib and SDRT induced ASMase activity up to 3.91 ± 0.55 mol/ul/h at 1 h and 2.61 ± 0.13 mol/ul/h at 6 h after radiation, respectively, significantly higher than Pazopanib alone (2.69 ± 0.32 mol/ul/h at 1 h and 1.9 ± 0.24 mol/ul/h at 6 h) or SDRT alone (2.78 ± 0.35 mol/ul/h at 1 h and 2.3 ± 0.11 mol/ul/h at 6 h) in MPNST3 tumors. Similar results were achieved in JJ012 sarcoma model. Combined therapy increased ASMase activity to 3.91 ± 0.45 mol/ul/h at 1 h and 2.6 ± 0.32mol/ul/h at 6 h post-SDRT, significantly higher than Pazopanib alone (2.69 ± 0.45 mol/ul/h at 1 h and 2.77 ± 0.56 mol/ul/h at 6 h) or SDRT alone (1.8 ± 0.6 mol/ul/h at 1 h and 2.29 ± 0.67 mol/ul/h at 6 h). Altogether these data indicate that the combination of SDRT with the short-acting anti-angiogenic agent, Pazopanib, generates a ceramide-driven vascular dysfunction resulting in significant tumor growth delay in two human sarcoma models.

### Pazopanib radiosensitized bovine aortic endothelial cells (BAEC) and increased SDRT-induced cell apoptosis *in vitro*

**Figure 5:**
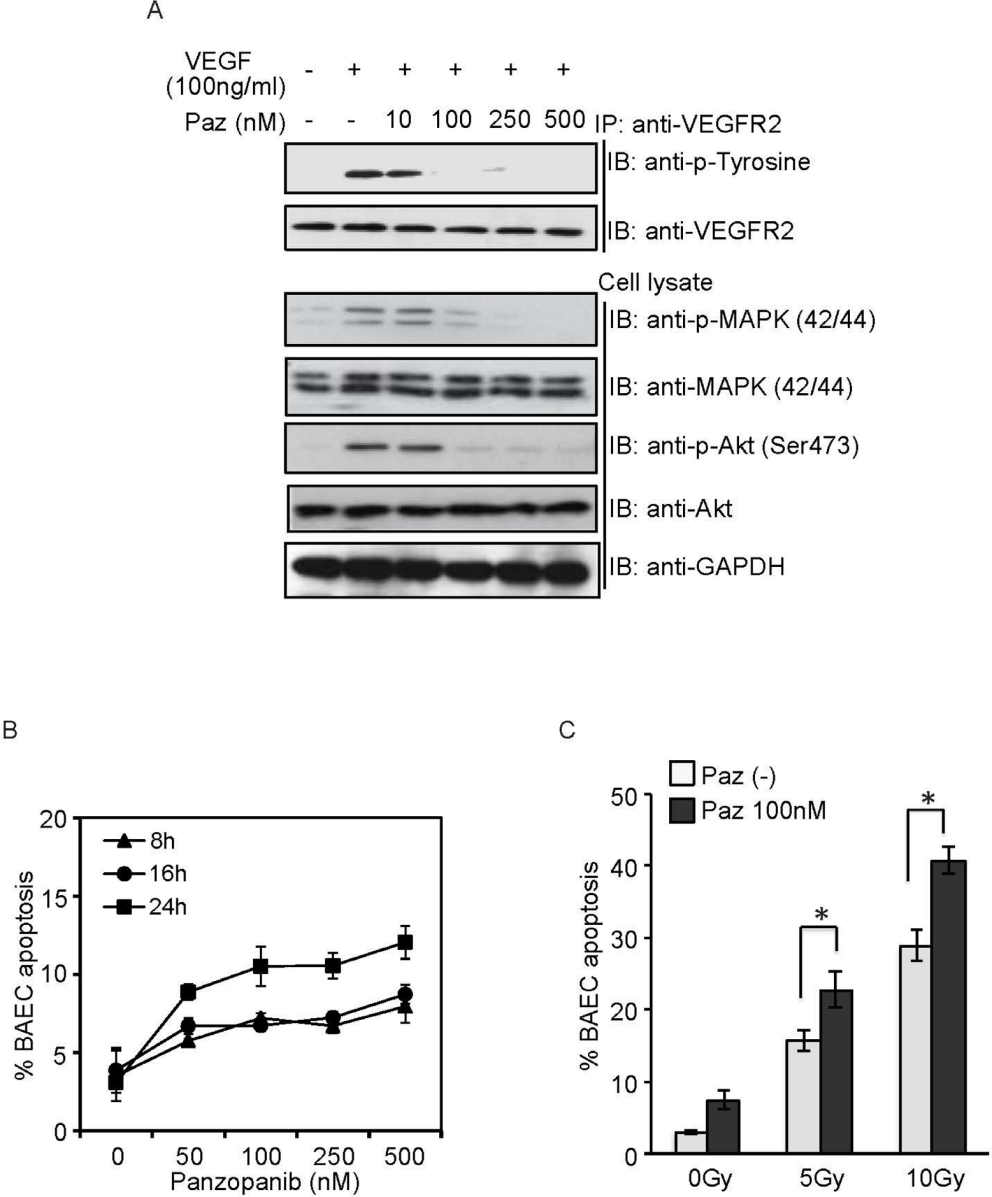
Pazopanib radiosensitized BAEC *in vitro* (**A**) BAEC were pre-treated with Pazopanib at the indicated doses for 1 h, and stimulated with VEGF (100 ng/ml) for 10 min. Cell lysate was analyzed by western blotting. Endogenous VEGFR2 were immunoprecipitated and analyzed by western blotting against anti-phosphotyrosine antibody. (**B**) BAECs were exposed to Pazopanib at the indicated dose and time. Cells were fixed, and apoptosis was assessed using bis-benzimide staining. (**C**) After pre-treatment with Pazopanib (100 nM) for 16 h, BAECs were irradiated at the indicated doses. Cells were fixed at 8 h post-radiation, and apoptosis was assessed using *bis*-benzimide staining. Each value represents the mean ± SD of duplicate determinations from three separate studies. ^*^ *p* < 0.05.

Angiogenic factors (bFGF and VEGF, etc) inhibited SDRT-induced EC apoptosis, although the underlying mechanism remains unclear [13, 14]. In order to demonstrate that the synergistic effect of Pazopanib results from inhibition of VEGF signaling we tested these effects on BAEC *in vitro*, which has been demonstrated to be an ideal model for radiosensitization to SDRT [24]. As shown in Figure 5A, 100 ng/ml VEGF elicited a rapid activation of VEGFR2 and 2 crucial downstream proteins, ERK and Akt in BAECs. Pazopanib at a low concentration (10 ng/ml) did not repress VEGF-induced phosphorylation of VEGFR2, Akt and Erk, whereas high concentration of Pazopanib (>100 ng/ml) showed a dramatic inhibition effect of all three, and 500 ng/ml Pazopanib led to a complete repression of phosphorylation of these targets. These results indicated that Pazopanib is a potent inhibitor of VEGF signaling in BAECs. Next, we assessed whether Pazopanib administration could induce BAEC apoptosis. Similar to our results with DC101 (antibody against VEGF - R2) [14], Pazopanib administration showed the best induction of apoptosis in BAEC at 24 h (Figure 5B). Based on these results we pre-incubated the cells with Pazopanib for 16 h and examined a dose response radiosensitization in BAECs. As shown in Figure 5C, a massive increase in BAEC apoptosis (15.8% ± 1.5% at 5 Gy and 28.9% ± 2.2% at 10 Gy) was achieved at 8 h post SDRT, whereas Pazopanib alone (100 ng/ml) induced a very minimal apoptotic effect. Importantly, substantially enhanced EC apoptosis (22.9% ± 2.5% at 5 Gy and 40.8% ± 1.9% at 10 Gy) was generated in the combined condition (Figure 5C), which was significantly higher than with each treatment alone. These results provided strong evidence that Pazopanib has a direct effect on the endothelial cells and synergizes with SDRT in their dysfunction.

### Combination of Pazopanib and SDRT increased ASMase activity and C_16_-ceramide level in BAECs

**Figure 6:**
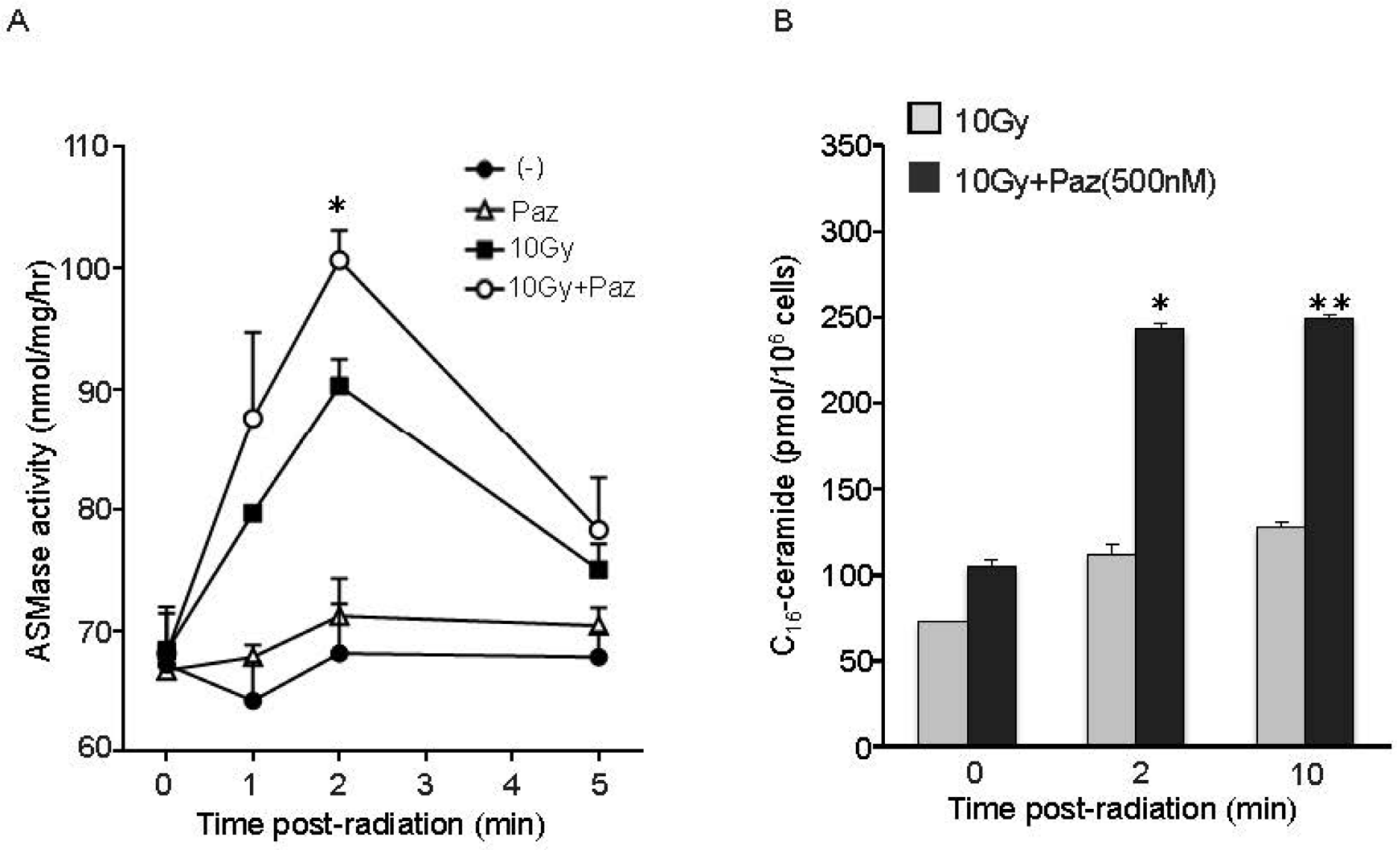
Pre-treatment with Pazopanib significantly increased SDRT-induced activation of ASMase and generation of ceramide levels in BAEC cells BAECs were irradiated at 10 Gy, followed by pre-treatment with Pazopanib for 16 h. Cells were collected at indicated time after radiation and ASMase activity was measured by quantifying conversion of (^14^C)sphingomyelin to the product (^14^C)phosphocholine. The result represents the duplicate determinations from 2 experiments. Data is the mean ± SD. ^*^ indicated *p* < 0.05 (10 Gy vs 10 Gy + Paz) (**A**). After pre-treatment with Pazopanib for 16 h, BAECs were irradiated at 10 Gy. Total lipids were extracted at the indicated time after radiation, and C_16_-ceramide level was measured by liquid chromatography, electrospray ionization-tandem mass spectrometry. Data is the representative determination from 2 experiments. Data is the mean ± SD. ^*^p < 0.05 and ^**^p < 0.01; 10 Gy vs 10 Gy + Paz (**B**).

In order to establish that the Pazopanib-induced radiosensitization is mediated via ASMase/ceramide pathway activation, we assessed ASMase activity and ceramide levels in BAEC after Pazopanib treatment in combination with SDRT. As shown in Figure 6A, 10 Gy radiation rapidly triggered an increase in ASMase activity in BAEC from a baseline of 68.34 ± 2.41 mol/mg/h to peak at 90.42 ± 1.84 mol/mg/h at 2 min after radiation, similar to our previously reported results in these cells. Pre-treatment of Pazopanib further elevated ASMase activity up to 101.78 ± 2.6 mol/mg/h at 2 min, which was significantly higher than radiation alone. Combination of Pazopanib and SDRT also enhanced the total ceramide content compared to Pazopanib or SDRT alone treatment (Table 1). Recent studies revealed that C_16_-ceramide was preferentially associated with stress-induced apoptosis in a variety of cell types [25–27]. In this study, C_16_-ceramide, the apoptogenic ceramide, level was immediately enhanced after radiation treatment, and increased from a baseline of 72.71 ± 0.47 pmol/10^6^ cells to 112.25 ± 5.88 pmol/10^6^ cells at 2 min and to 127.36 ± 3.04 pmol/10^6^ cells at 10 min (Figure 6B and Table 1). Pazopanib induced a significant increase in the generation of ceramide (243.03 ± 3.65 pmol/10^6^ cells) at 2 min, which was maintained over 10 min (249.48 ± 1.8 pmol/10^6^ cells, *p* < 0.05% vs SDRT alone). There were no significant differences in the other ceramide species levels in response to Pazopanib or SDRT alone or in their combination. These results indicate that Pazopanib potentiates radiation-induced apoptosis in endothelial cells via modulation of ASMase/ceramide signaling.

**Table 1:** Ceramide levels in BAEC following treatment with Pazopanib and RT

*Ceramide pmol/10^6^ cells*	Treatment					
	(−)	Paz	10 Gy (2 min)	Paz + 10 Gy (2 min)	10 Gy (10 min)	Paz + 10 Gy (10 min)
***C14:0***	4.791 ± 0.478	6.594 ± 3.754	7.269 ± 1.459	7.847 ± 0.371	8.046 ± 0.943	8.181 ± 1.019
***C16:0***	72.711 ± 0.465	105.087 ± 3.94	112.247 ± 5.884	243.025 ± 3.65	127.364 ± 3.044	249.478 ± 1.795
***C18:1***	22.561 ± 0.651	44.899 ± 1.82	33.859 ± 2.073	68.533 ± 3.389	31.763 ± 2.636	54.661 ± 1.251
***C18:0***	5.524 ± 0.612	11.619 ± 0.675	7.33 ± 0.848	19.395 ± 1.798	6.8 ± 3.33	13.585 ± 1.607
***C20:0***	0.798 ± 0.256	1.44 ± 0.242	0.721 ± 0.389	1.804 ± 0.743	1.033 ± 0.274	1.646 ± 0.619
***C22:0***	6.737 ± 0.589	10.503 ± 1.002	9.458 ± 1.618	19.987 ± 1.797	9.963 ± 0.216	15.55 ± 1.862
***C24:1***	22.045 ± 0.601	38.391 ± 2.269	29.812 ± 2.55	43.279 ± 1.846	32.918 ± 1.61	47.941 ± 1.127
***C24:0***	14.238 ± 1.26	17.044 ± 4.438	20.416 ± 1.49	35.374 ± 0.758	19.184 ± 1.593	28.275 ± 4.109
***C26:1***	2.934 ± 0.798	4.594 ± 1.819	5.179 ± 0.57	10.174 ± 1.826	4.593 ± 1.442	7.558 ± 1.099
***C26:0***	0.513 ± 0.097	1.07 ± 0.451	0.927 ± 0.486	1.692 ± 0.192	0.811 ± 0.248	1.19 ± 0.429

BAEC were irradiated at 10 Gy after pre-treatment with Pazopanib for 16 h. Total lipids were extracted at the indicated time post radiation, and ceramide content was measured by liquid chromatography, electrospray ionization-tandem mass spectrometry. Value is the mean ± SD. Paz: pazopanib; RT: radiation treatment.

## DISCUSSION

In this study, the molecular mechanism of combination of anti-VEGF reagent and SDRT on tumor response via amplification of ceramide-driven endothelial apoptosis was investigated in two human sarcoma animal models. Our findings revealed that pre-treatment with Pazopanib prior to SDRT enhances tumor endothelial dysfunction and tumor growth delay in these tumors. The results also confirm that the tissue VEGF confers resistance to radiotherapy, and provide a pre-clinical basis to the use of Pazopanib as an SDRT sensitizer to synergistically improve local tumor response.

Emerging evidence indicates that SDRT, such as used in this study, may provide therapeutic benefit to some human tumors, even to those considered resistant to conventional fractionated radiotherapy schemes and chemotherapy. Early clinical studies demonstrated that 24 Gy SDRT alone can locally cure >90% of human cancer regardless of tumor type or size, however this curative effect can be acquired only in cases where normal tissues could be completely avoided [4, 28, 29]. Thus, SDRT use is limited in many clinical settings by close proximity of the tumor to critical normal tissue. According to this principle, the critical dose limitation toxicity for SDRT in multiple clinical settings appears to generally be within the range of 14–16 Gy. Therefore, the challenge is to improve tumor response to lower doses of SDRT, and radiosensitization via temporally constrained use of anti-VEGFR agents may be the answer. Prior work demonstrated that bursts of free radical species, generated by waves of hypoxia/reoxygenation occurring after each radiation exposure, lead to induction of hypoxia-inducible factor-1α (HIF-1α) activity [30]. The up-regulation of HIF-1α elevates overexpression of VEGF and other pro-angiogenic factors, which protect tumor endothelium and confer radioresistance [30]. A recent report from a group at MSKCC showed that a hypoxia-activated chemotherapeutic TH-302 combined with VEGF-A inhibition and RT, an exploratory tri-modality therapy, dramatically enhanced tumor response in preclinical models of sarcoma via increasing DNA damage and apoptosis in endothelial cells and decreasing HIF-1α activity [31]. These results indicate that an anti-VEGF approach may improve the radiosensitization and increase local tumor response. Consistent with our previous publications, our current results further provide direct and clear evidence that anti-VEGF treatment synergistically improved tumor response through radiosensitization.

SDRT induced *in vivo* and *in vitro* endothelial cell apoptosis via activation of ASMase/ceramide signaling, which contributes to tumor growth delay or cure [6, 9, 14]. Within these studies, SDRT triggered TEC apoptosis as early as 2 h and peaked at 6–8 h post radiation treatment [6, 14]. Our results showed that SDRT drove an increase of *in vivo* TEC and *in vitro* BAEC apoptosis at 6–8 h post RT, which supported these findings. Tumor vasculature has been shown to be largely affected by RT depending on the number of fractions, dose rate, and total radiation dose [32]. SDRT can induce rapid apoptosis of TEC, whereas the importance of vascular damage in tumors receiving conventionally fractionated radiation therapy (CFRT: 1.8–2 Gy per fraction) is more controversial [6, 7, 30]. A recent study suggested that 2 Gy irradiation dose induced minimal endothelial cell apoptosis followed by a later increase in vessel diameter, microvascular density and vessel leakiness in normal brain blood vessels [33]. However, few reports so far provided clear proof that CRFT induced TEC apoptosis. Thus, the biological mechanism underlying fractionated radiotherapy may greatly differ from SDRT used in this study, which mainly functions via ASMase/ceramide-mediated vascular dysfunction.

As a generic mediator of stress, the ASMase/ceramide pathway has been shown to function on trans-activating pathogenesis of tissue damage in multiple models of human disease [34]. Endothelial cells are particularly sensitive to SDRT-induced apoptosis *in vitro* and *in vivo* because they have 20-fold higher levels of secretory ASMase relative to other mammalian cells [5, 10, 35]. Our results showed that SDRT launched ASMase activity *in vivo* and *in vitro*, which resulted in a significant increase in endothelial cell apoptosis via enhanced ceramide generation. Although the detailed mechanism by which radiation-induced translocation/activation of ASMase was not addressed here, the accumulated information over the last decade sheds light on this event. Diverse stresses (UV, IR etc) induce ASMase trafficking to the outer leaflet of the plasma membrane, where it converts sphingomyelin to ceramide [9, 36]. This process requires intact microtubules, phosphorylation of Ser508 of ASMase, as well as functional lipid rafts [37–39]. However, the mechanism by which VEGF may repress ASMase activity remains unclear. What is becoming clear from this and our other most recent studies (14) is that anti-VEGF pathway inhibitors synergistically increased SDRT-induced ASMase activity by reversing the effect of VEGF. In addition, several publications revealed that upregulation of ASMase occurred in diverse pathological conditions, and proposed that a significant change of serum ASMase may appear as a novel biomarker in disease [40, 41]. Sathishkumar *et al.* showed that serum ASMase activity and ceramide content increase following Spatially Fractionated high dose Radiation Treatment (SFGRT) [42] and correlate with the clinical response [43]. Here we also showed that SDRT promoted an increase of ASMase activity in mouse serum at 1 h and maintained high level at 6 h post-SDRT, and an anti-VEGF agent synergistically increased this activity. These results indicate that the changes of ASMase activity can serve as a biomarker when monitoring the delivery of SDRT to cancer patients. We have already confirmed this concept in patients in ongoing clinical trials at MSKCC (Campagne and Fuks, submitted).

Activated ASMase converts sphingomyelin to ceramide in the outer plasma membrane and leads to formation of ceramide-rich macrodomains (CRMs), which serve as sites of stress-related specific multiprotein complexes and relay downstream apoptotic signaling [9, 36]. We showed that SDRT induced a rapid increase of the C_16_-ceramide (Table 1), which is the apoptogenic ceramide species, and Pazopanib synergistically increased the C_16_-ceramide level. The increase of C_16_-ceramide contributes to the reorganization of membrane rafts into large signaling platforms, CRMs, which affords a mechanism by which SDRT induces endothelial apoptosis and generates microvascular dysfunction. Recently, increasing studies using LC-MS to determine the changes in specific ceramide species revealed that C_16_-ceramide was preferentially associated with stress-induced apoptosis in a variety of cell types. C_16_-ceramide has also been shown to increase the sensitivity of Jurkat T cells and hepatocytes [25], and human chronic myelogenous leukemia (CML) [26] to Fas-mediated apoptosis. Increases in C_16_-ceramide levels in Jurkat cells occurred 2 h after initiation of apoptosis by ionizing radiation, whereas no change of C_16_-ceramide level was observed in RT-resistant cells. In the current study, C_16_-ceramide levels rapidly increased 2 min after SDRT and remained elevated for more than 10 min. The high radiosensitivity of BAEC may result from the 20–fold higher ASMase level in these cells as compared with other cells in the body. In addition to its crucial role in the formation of CRMs to mediate apoptotic signaling, C_16_-ceramide may also induce apoptosis by repression of pro-survival pathways. C_16_-Ceramide has been shown to activate ceramide-activated protein phosphatase leading to the dephosphorylation of p38 and Akt, which may negatively regulate the activities of downstream factors implicated in the regulation of apoptosis [27, 44].

Recently, Booth *et al.* reported that Pazopanib combined with PDE5i or ERBB1/2/4 inhibitor (afatinib) induced tumor cells (fresh PDX isolate of NSCLC) death via ER-induced stress by toxic autophagy and by affecting the chaperone activity in these cells an induction of ER stress-induced autophagy death [45]. The ER stress-induced autophagy death is mediated via the pERK/eIF2α pathway. Furthermore, they also showed that pazopanib and BYL719 combination killed HCT116 cells that express a mutated active K-RAS protein and an activated PI3K-AKT-mTOR signaling pathway [45] indicating that pazopanib-dependent activation of the pERK/eIF2α pathway might be stress- and cell type-specific. Relative to these findings, our study focused on optimizing the effects of RT that induce microvascular dysfunction. Microvascular cells do not overexpress chaperones, a characteristic specific to tumor cells, or other mutation such as K-RAS, nor do they have constitutively active signaling pathways that drive proliferation. Therefore, the pERK/eIF2α pathway-mediated autophagy death may not occur in tumor-associated endothelial cells. The direct impact of SDRT and/or pazopanib on tumor cells, will be investigated in a future study.

The data in this study also indicated that pre-treatment with anti-VEGF agent 1 h prior to SDRT is required for anti-angiogenic de-repression of endothelial ASMase and consequent tumor response. This result supported our previous findings that 1 h prior to RT is the optimal time window for treating tumors using anti-angiogenic agents in combination with SDRT [14, 23]. Notably, this temporal relationship between anti-angiogenic agents and radiation differs from the tumor microvessel normalization hypothesis, which requires at least 24 h to manifest, or constraint of endothelial progenitor recruitment into the damaged site, usually delivered at 24 h prior to irradiation [18, 19]. Although the underlying mechanism remains elusive, given that SDRT increased ASMase activity and ceramide generation just within minutes, it is reasonable to assume that anti-angiogenic agents should be delivered immediately prior to irradiation to de-repress ASMase.

In summary, recent understanding of the effects of the synergistic inhibition of short-acting anti-angiogenic agents and SDRT on tumor endothelium provides new targets for improving local cure of human cancer with radiation. The conclusion from this study, that a single dose of Pazopanib substantially sensitizes tumors to the effects SDRT, indicates this as a highly promising treatment strategy for sarcomas resistant to conventional RT and chemotherapy.

## MATERIALS AND METHODS

### Materials

Pazopanib was purchased from GlaxoSmithKline (GSK). Bis-benzimide was purchased from Life Technology (Carlsbad, CA). Anti-VEGFR2 (FLK) was purchased from Santa Cruz Biotech. (Santa Cruz, CA). Anti-Cleaved Caspase3, anti-Meca32, Anti-Akt, anti-Phospho-Akt (Ser473), anti-Erk, anti-Phospho-Erk and anti-GAPDH were purchased from Cell Signaling Technology (Beverly, MA). Protein G Sepharose beads were purchased from GE Health Biosciences GE Healthcare (Piscataway, NJ). [14C-methylcholine] sphingomyelin was purchased from Amersham Biosciences (Piscataway, NJ).

### Cell culture

Bovine aortic endothelial cells (BAEC), established from the intima of bovine aorta as described [24], were maintained in DMEM supplemented with 5% calf serum, 100 U/ml penicillin, 100 μg/ml streptomycin, and 2 mM L-glutamine at 37° C in a humidified 10% CO_2_ chamber. JJ012 cell line and MPNST3 tumors given to us by the Singer lab at Memorial Sloan Kettering Cancer Center cells were maintained in DMEM containing glucose (4.5 g/L), glutamine (2 mM), penicillin (50 U/ml), and streptomycin (100 mg/ml) supplemented with 10% fetal bovine serum in a humidified 5% CO_2_ chamber.

### Animal experiments

All the animal experiments were performed according to the guidelines, following a protocol approved by the Institutional Animal Care and Use Committee (IACUC). ICR-SCID mice, 6–8 week-old, were purchased from Jackson laboratory (Bar Harbor, ME); athymic nu/nu mice, 6–8 week-old, were purchased from Envigo (Indianapolis, IN) and housed at the Research Animal Resource Center (RARC) of Memorial Sloan-Kettering Cancer Center. The facility is approved by the American Association for Accreditation of Laboratory Animal Care and is maintained in accordance with the regulations and standards of the United States Department of Agriculture and the Department of Health and Human Services, NIH.

Neurofibrosarcoma (MPNST3) tumor tissue was transplanted subcutaneously into the right flank of ICR-SCID mice and chondrosarcoma (JJ012) cells were implanted subcutaneously into the right flank of athymic mice (15 million cells/mouse). Once tumors reached a size of 100–150 mm^3^, mice were either treated with SDRT and/or Pazopanib (100 mg/kg, orally). Radiation was delivered using a Philips MG-324 X-ray at 117 cGy/min (50 cm source to skin distance). Mice were lightly sedated with ketamine (0.1 mg/g) and xylazine (0.02 mg/g) and only tumor, surrounding skin and subcutaneous tissues were exposed using a specialized lead jig. Tumor volumes, based on caliper measurements, were calculated daily using the formula of V = (W^2^ × L)/2 [46].

### ASMase activity assay

ASMase activity was measured by radioenzymatic assay using [^14^C-methylcholine] sphingomyelin as substrate, as described with minor modifications [8]. Briefly, following treatment with different conditions, cells were washed with ice cold PBS and lysed in PBS containing 0.2% Triton X-100. 2.5 ug cell lysate were incubated with a total 9.5 nmol sphingomyelin substrate mixture including 0.026 μCi[^14^C-methylcholine] sphingomyelin in reaction buffer (250 mM sodium acetate, pH 5.0 supplemented with 0.1% Triton X-100 and 1 mM EDTA) at 37° C for 2 h. Reactions were terminated by adding 125 ul of mixture of CHCl3:MeOH:HCl, 100:100:1 v/v/v, and the upper phase was removed and quantified by a Beckman Packard 2200 CA Tricarb scintillation counter.

### Apoptosis assay

Fluorochrome *bis*-benzimide trihydrochloride (Hoechst-33258) was used to visualize the morphologic changes of nuclear chromatin in cells undergoing apoptosis as described [35]. Briefly, following treatment BAECs including floating cells were harvested and fixed with 4% paraformaldehyde, washed with phosphate buffered saline (PBS) and stained with 50 μl of 24 μg/ml *bis*-benzimide trihydrochloride solution for 10 minutes. Apoptotic cells were quantified using an Axiovert S-100 Zeiss epifluorescent wide-field microscope equipped with a DAPI filter set. A minimum of 500 cells was examined per point.

*In vivo* tumor endothelial cell (TEC) apoptosis was measured by double staining with anti-cleaved caspase 3, to detect apoptotic cells, and the endothelial cell surface marker MECA-32, to identify tumor endothelium. Briefly, tumor specimens were removed after SDRT and/or Pazopanib treatment at the indicated time points, fixed in 4% paraformaldehyde, embedded in paraffin, and 5-μm sections were stained. Red-Green–Blue–merged endothelial cell was counted as an apoptotic cell, and a minimum of 500 endothelial cells were evaluated per point.

### Mass spectrometric analysis of ceramide

After appropriate treatment, cells were incubated at 37° C for the indicated time and reaction was stopped by placing the cells on ice. Floating cells were collected in chilled 13 × 100 mm glass tubes by centrifugation at 500 g for 5 min. Cells were washed once with cold PBS, and lipids were extracted by incubating cells in 0.5 ml of CH_3_OH for 10 minutes. Cell lysates were then added to the pelleted floating cells. Ceramide levels were analyzed using liquid chromatography, electrospray ionization-tandem mass spectrometry as described previously [47].

### Microvessel density (MVD) measurement

Removed tumor tissues were fixed in 4% paraformaldehyde, and embedded in paraffin. 5-mm sections of tumor specimens were stained with MECA-32 [41] to detect TECs. Microvessel density was quantified using MetaMorph image analysis software and MECA-32^+^ [41] area was calculated in each section.

### Perfusion measurement in the tumor

Mice were injected with the fluorescent dye Hoechst 33342 (Tocris Bioscience), 20 mg/kg, 30 minutes after irradiation. Mice were sacrificed 2 minutes after injection and tumors were frozen on dry ice in O.C.T. compound (Scigen), cryosectioned and fixed with 4% paraformaldehyde. Tumor sections were imaged using Pannoramic 250 Flash digital slide scanner (3D Histech, Hungary) and fluorescence was quantified using ImageJ software [48].

### Western blotting and immunoprecipitation

Western blotting and immunoprecipitation were performed as described previously [49].

### Statistics

Statistical analysis was performed using Graph Pad Prism 6.0. A two-tailed Student *t* test was used to compare the mean values between two groups. *P* < 0.05 was considered to be significant.

## Abbreviations

SDRT: single high dose radiation therapy
ASMase: Acid Sphingomyelinase
VEGF: vascular endothelial growth factor
RT: radiation therapy
IR: Ionizing radiation
BAEC: bovine aortic endothelial cells
PBS: phosphate buffered saline
TEC: tumor endothelial cell
MVD: Microvessel density
HIF-1: hypoxia-inducible factor-1
CFRT: conventionally fractionated radiation therapy
CRMs: ceramide-rich macrodomains
CML: chronic myelogenous leukemia.
